# MicroRNA-33a negatively regulates myoblast proliferation by targeting IGF1, follistatin and cyclin D1

**DOI:** 10.1042/BSR20191327

**Published:** 2020-06-04

**Authors:** Xinxin Li, Jiamin Qiu, Hehe Liu, Yan Deng, Shenqiang Hu, Jiwei Hu, Yushi Wang, Jiwen Wang

**Affiliations:** 1 Division of Farm Animal Genetic Resources Exploration and Innovation, Key Laboratory of Sichuan Province, College of Animal Science and Technology, Sichuan Agricultural University, Chengdu, Sichuan, Public Republic of China, 611130; 2 Institute of Medical Research, Northwestern Polytechnical University, Xi'an, Shaanxi, Public Republic of China, 710072

**Keywords:** CCND1, FST, IGF1, miR-33a, myoblast proliferation, PI3K/Akt/mTOR signaling pathway

## Abstract

MiR-33a is found as a regulator of cell proliferation in many cancer cells. However, it remains unknown if and how miR-33a plays a role in myoblast proliferation. To investigate the effect of miR-33a on myoblast proliferation, miR-33a mimic or inhibitor was co-administered with or without insulin-like growth factor 1 (IGF1) to simulation myoblasts. Our study showed that up-regulation of miR-33a impaired myoblast proliferation, while down-regulation of miR-33a enhanced myoblast proliferation. Mechanistically, we examined that miR-33a can inhibit the transcription of IGF1, follistatin (FST) and cyclin D1 (CCND1) by targeting their 3′UTR region in both HEK293T cells and duck myoblasts. Moreover, up-regulation of miR-33a decreased and its down-regulation increased the mRNA expression of PI3K, Akt, mTOR and S6K. Importantly, the decreased PI3K, Akt, mTOR and S6K expression by miR-33a mimics was abrogated by co-administered with IGF1. Altogether, our results demonstrated that miR-33a may directly target IGF1, FST and CCND1 to inhibit myoblast proliferation via PI3K/Akt/mTOR signaling pathway. In conclusion, miR-33a is a potential negative regulator of myoblast proliferation and by modulating its expression could promote the early development of skeletal muscle.

## Introduction

The development of skeletal muscle (myogenesis) is a multi-step process. Specifically, the multi-potent precursor cells commit into myoblasts followed by proliferation and withdrawal from the cell cycle. Myoblasts further differentiates and fuses into multinucleated myotubes, which finally ends up with myofibres formation [1]. During this highly orchestrated process, myoblast proliferation is an early cellular event that is critical for myogenesis [2]. The regulation of myoblast proliferation requires many regulators, such as growth factors, transcription factors and miRNAs. It has been reported that a subset of miRNAs participate in the regulation of myoblast proliferation in patients with muscular dystrophy, mouse or pig [3] and many miRNAs have been identified as critical regulators of early myogenesis [4].

miRNAs are small non-coding RNAs with the length of approximately 22 nucleotides, and they generally normally act as negative regulators of the post-transcriptional expression of genes [5]. Previous studies suggested that both muscle-specific and its nonspecific miRNAs are important in myogenesis. Several muscle-specific miRNAs, such as miR-1, miR-133a/b and miR-206, are key regulators in human myogenesis [6]. Muscle-nonspecific miRNAs, such as miR-27b and miR-29, may also contribute to myogenesis and affect skeletal muscle growth and hypertrophy [7,8].

miR-33a, a muscle-nonspecific miRNA, is a member of the miR-33 family, which is highly conserved from drosophila to humans [9]. It has been shown that miR-33a is an important regulator of many cellular processes including cell cycle, proliferation, insulin signaling and glucose homeostasis. Overexpression of miR-33a inhibits cell proliferation in human melanoma SK-MEL-1 and WM-115 cell lines [10]. In murine hepatocytes, miR-33a can directly target the 3′UTR (untranslated region) of CCND1 and cyclin-dependent kinase 6 (CDK6) to inhibit cell proliferation [11]. It has been reported that overexpression of miR-33a inhibits the activation of insulin signaling pathway and regulates insulin sensibility in Huh7 cells [12,13]. However, the role of miR-33a in regulation of skeletal muscle development has not been described.

It is well known that IGF1, CCND1 and FST play a central role in regulating skeletal muscle growth by stimulating myoblast proliferation. Specifically, IGF1 is an important growth factor, and some researches showed that the knockout of IGF1 gene in mice will induce skeletal muscle atrophy by inhibiting myoblast proliferation [14,15]. CCND1 is a pivotal cell cycle regulator, and it promotes the proliferation of myoblasts by regulating myogenic differentiation antigen (MyoD) [16]. Down-regulation of CCND1 induces cell cycle arrest [17]. FST is an important secretory protein, and its deficiency leads to perinatal death in mice [18] whereas its transgenic form or postnatal overexpression induces muscle hypertrophy and myoblast proliferation [19,20]. By using targetscan software, IGF1, FST and CCND1 were predicted to be the target genes of miR-33a. Based on the function of these genes, we hypothesized that miR-33a might be a potential negative regulator in myogenesis. In the present study, we revealed the effects of miR-33a on duck myoblast proliferation, which provides insights into miR-33a function in the overall process of skeletal muscle development and its potential therapeutic use in skeletal muscle hypertrophy.

## Materials and methods

### Animals

Thirteen-day-old duck eggs were provided by the Sichuan Agricultural University Waterfowl Breeding Experimental Farm located in Ya’an, China. All the eggs were incubated under the same conditions at 37 ± 0.5°C with the humidity of 70 ± 5%. All procedures in the present study were conducted in compliance with the recommendations in the Guide for Sichuan Agricultural University Animal Care and Use Committee, Sichuan Agricultural University, Sichuan, China.

### Isolation and culture of primary myoblasts

Primary myoblasts were isolated from the leg muscles taken from 13-day-old duck embryos according to the method described previously by Liu et al. [20,21]. The myoblasts were cultured in growth medium containing Dulbecco’s modified Eagle’s medium (DMEM) (Hyclone, U.S.A.) with 10% fetal bovine serum (FBS) (Gibco, U.S.A.) and penicillin (100 U/ml) and streptomycin (100 µg/ml). The myoblasts were cultured in an incubator at 37°C with 5% CO_2_.

### Cell transfection and treatment

The myoblasts were transfected with different concentrations of miR-33a mimics (25, 50 and 100 nM) or inhibitors (50, 100 and 200 nM) by using Lipo2000 transfection reagent (Beyotime, China) according to the manufacturer’s instruction. All experimental control samples were treated with an equal concentration of a non-targeting control mimic sequence or inhibitor negative control sequence to control for non-sequence specific effects in experiments. After 12, 24 and 48 h of incubation, the cells were collected for subsequent analyses. In the treatment and transfection assays involving IGF1, the myoblasts were first treated with IGF1 protein for 12 h. Then, the cells were washed twice with PBS and transfected separately with miR-33a mimics or miR-33a inhibitors for 24 h. The cells were harvested for analyses at the end of each incubation period.

### Cell viability assay

Myoblasts were seeded at a density of 0.5 × 10^4^ cells/well in a 96-well plate, and the viability of myoblasts was evaluated by using the MTT [3-(4, 5-dimethylthiazol-2yl) -2,5-diphenyltetrazolium bromide] method. After transfecting with miR-33a mimics or miR-33a inhibitors for 12, 24 and 48 h, 10 μl of MTT (5 mg/ml, BiYunTian Biotechnology, China) was added to the 96-well plates for 4 h. The supernatants were then removed and 100 μl formazan (BiYunTian Biotechnology, China) were added to each well. At the end of the incubation period, the absorbance in each well was measured at 570 nm using a microplate reader (Thermo Fisher, U.S.A.).

### BrdU (5-bromo-2V-deoxyuridine) assay and immunofluorescence

The BrdU assay was performed as described previously [20]. The myoblasts were incubated for 4 h at 37°C in 25 μM BrdU (10 mg/ml in PBS, Boster). Blocking was achieved using a blocking solution [1% (w/v) BSA in PBS] for 30 min. The anti-BrdU antibody diluted 1:10 in PBS (Solarbio, China) was added to the wells and incubated overnight at 4°C. The cells were washed with PBS for three times and incubated with a goat anti-mouse IgG antibody (Boster,China) diluted 1: 100 in PBS for 2 h at 37°C. The nuclei were labeled with DAPI (4′, 6-diamidino-2 phenylindole; 10 μg/ml in PBS; BiYunTian Biotechnology, China). The cells were inspected under a fluorescence microscope (Nikon), and the images were analyzed using the Image-Pro plus 6.0 software (Media Cybernetics).

### Quantitative real-time PCR

Total RNA was isolated using Trizol reagent (Takara, Japan) according to the manufacturer’s protocol (the specific protocol is in the Supplementary Materials and Methods). RNA concentration was measured using a spectrophotometer. SYBR Prime Script RT-PCR Kit (TaKaRa, Japan) and Bio-Rad CFX Manager (Bio-Rad Laboratories, U.S.A.) were used for quantitative RT-PCR. The samples were tested in triplicates. The relative expression of target genes was normalized against the internal control genes *GADPH* (AY436595) and *β-actin* (EF667345), *U6* was used as the reference gene for miR-33a. The relative gene expression was determined by using the comparative Ct (2-^ΔΔ^Ct) method [22]. Primer sequences used for quantitative RT-PCR are listed in Supplementary Table S1.

### Western blot

Total cellular proteins were extracted from the myoblasts using RIPA lysis buffer (Beyotime, China). Protein samples were resolved on 10% SDS-PAGE gels and electroblotted on polyvinylidene difluoride (PVDF) membranes. The membranes were incubated in blocking buffer for 2 h at 37°C and then incubated in primary antibody for 12 h at 4°C. The membranes were incubated in secondary antibody for 2 h at 37°C. The protein bands were visualized using the ECL kit and a gel imaging system (Bio-Rad, U.S.A.). The anti-Akt, anti-phospho-Akt (Thr308), anti-mTOR, anti-S6K, anti-phospho-S6K (Ser417) and anti-tubulin rabbit monoclonal antibodies were purchased from Beijing Biosynthesis Biotechnology (Beijing, China). Anti-phospho-mTOR (serine 2448) rabbit monoclonal antibody was purchased from Cell Signaling Technology, U.S.A. Anti-tubulin antibody, used as a control, as well as the secondary antibodies (HRP-conjugated goat anti-rabbit IgG or goat anti-mouse IgG) was purchased from Biosynthesis Biotechnology (Beijing, China). All antibodies were diluted 1:1000 before use.

### 3′UTR luciferase reporter assays

For the luciferase reporter assay, HEK293T cells (ATCC, U.S.A.) and duck primary myoblasts were plated in 24-well plates (2 × 10^4^ cells/well) for 24 h. psiCHECK2-IGF1, psiCHECK2-FST, psiCHECK2-CCND1 or psiCHECK™-2 were co-transfected with 50 nM of miR-33a mimics, or negative controls (RiboBio, China). After transfection for 72 h, the cells were lysed in passive lysis buffer (Promega) and activities of firefly and renilla luciferase were measured with a GloMax 20/20 Luminometer (Promega) using the dual-luciferase reporter assay system (Promega) according to the manufacturer’s instructions. Primer sequences used to amplify 3′UTR of IGF1, FST and CCND1 are listed in Supplementary Table S2.

### Bioinformatics and statistical analysis

miR-33a target sites were predicted by using TargetScan 6.2 [23]. Differences in relative gene expression levels were analyzed by using ANOVA. The means were compared for significance using Tukey’s test. All results are expressed as mean ± SEM. The ANOVA and t-test were performed using SAS (SAS Institute, Cary, NC, U.S.A.). *P* values less than 0.05 were considered statistically significant.

## Results

### Validation of gain and loss of function experiments in duck primary myoblasts

**Figure 1:**
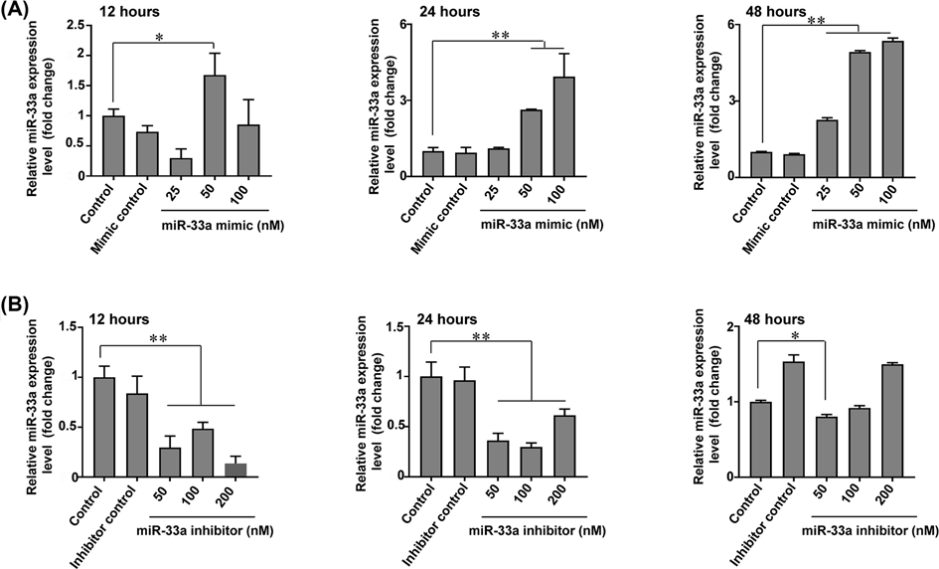
miR-33a mimics and miR-33a inhibitors are successfully transfected into myoblasts (**A**) Quantitative RT-PCR analysis of miR-33a expression in myoblasts after transfection with different concentrations of miR-33a mimics for 12, 24 and 48 h. Data were analyzed by the Student’s *t* test and shown as mean ± SEM; *N*=3, **P<0.01, *P<0.05. (**B**) Quantitative RT-PCR analysis of miR-33a expression in myoblasts after transfection with different concentrations of miR-33a inhibitors for 12, 24 and 48 h. Data were analyzed by the Student *t* test and shown as mean ± SEM; *N*=3, **P<0.01, *P<0.05.

In order to explore the function of miR-33a in myoblasts, firstly, we identified the mRNA expression of miR-33a in myoblasts. The result showed that the expression of miR-33a was highest at 0 h and lowest at 48 h (Supplementary Figure S1). Secondly, to ensure that miR-33a mimics or inhibitors are successfully transfected into myoblasts, the overexpression and interference efficiency of miR-33a was measured. We detected the expression level of miR-33a at 12, 24, and 48 h after transfection with miR-33a mimics or miR-33a inhibitors. The results showed that the three different doses of miR-33a mimics were successfully transfected into myoblasts and the transfection efficiency of miR-33a mimic in 24 h (100nM) and 48 h (50 nM/100 nM) groups are significantly higher than other groups (Figure 1A). Among all groups, the three doses of miR-33a inhibitors were successfully transfected into myoblasts in 12 and 24 h (Figure 1B). The results demonstrated that, with the increasing of transfection time, the transfection efficiency of miR-33a mimics gradually increased, but the transfection efficiency of miR-33a inhibitors gradually decreased.

### miR-33a impairs viability and inhibits the proliferation of myoblasts

**Figure 2:**
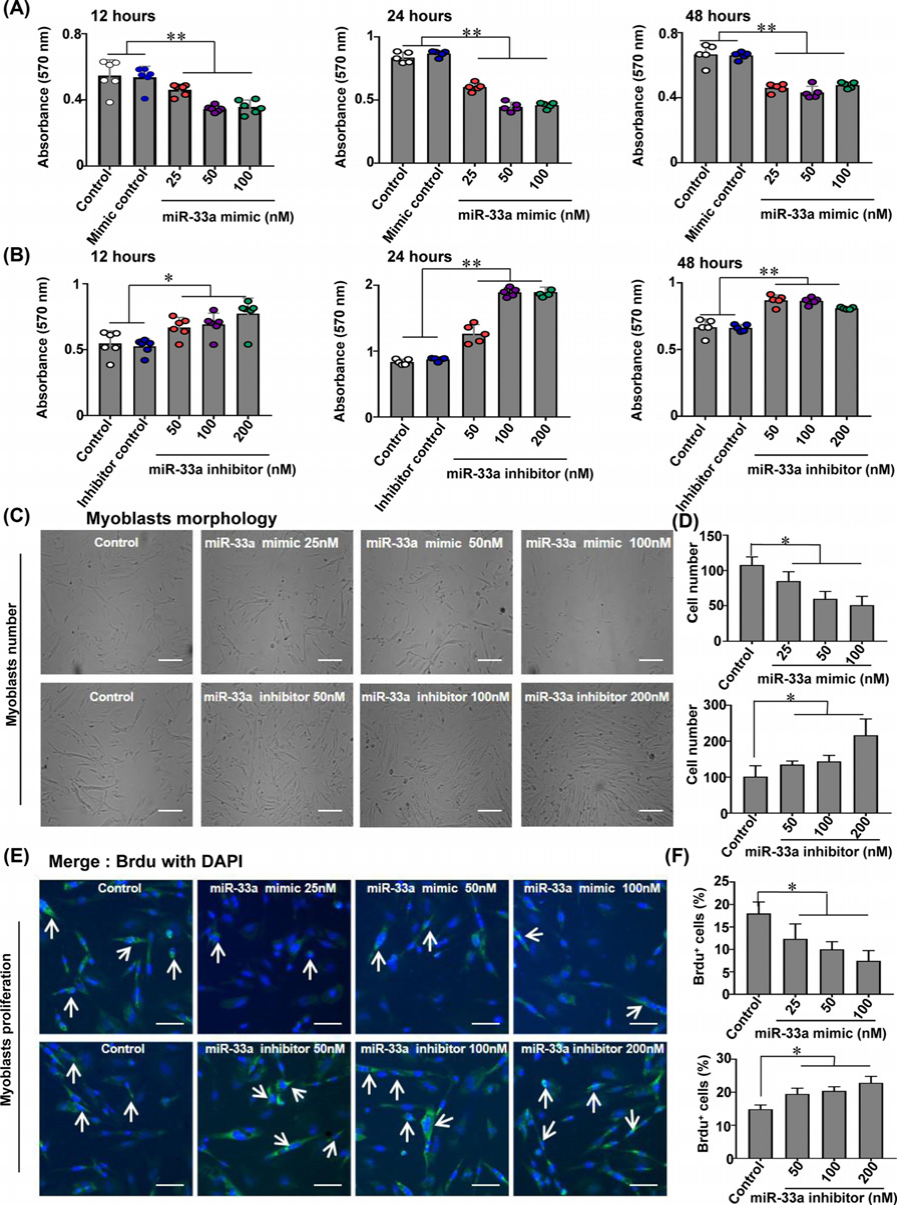
miR-33a reduces viability and proliferation of myoblasts (**A** and **B**) Myoblast proliferation was measured by MTT assay. (**C** and **D**) Representative images of myoblasts and cell count statistics in different treatment groups (100×). (**E** and **F**) Representative images of BrdU-labeled myoblasts and cell count statistics for different treatment groups; all of the nuclei are labeled blue by DAPI, but only the proliferating nuclei are labeled green by monoclonal anti-BrdU (200×), the arrows points the proliferation myoblasts. Data were analyzed by the Student *t* test and shown as mean ± SEM; *N*=3–5 wells; **P<0.01; *P<0.05.

To investigate the effect of miR-33a on myoblasts, we determined viability in myoblasts after treatment with miR-33a mimics or inhibitors. Overexpression and inhibition of miR-33a enhanced and diminished myoblasts viability, respectively, in spite of different concentrations. Specifically, the cell activity in miR-33a mimic 24 h (100 nM) group is lower than miR-33a mimic 48 h (50 nM/100 nM) groups, and the cell activity of miR-33a inhibitor 24 h (100 nM) group is higher than other groups (Figure 2A,B). Consistently, the number of myoblasts decreased with the increase in miR-33a mimic concentrations, but increased with the increase in miR-33a inhibitor concentrations significantly (Figure 2C,D). Additionally, the percentage of BrdU-labeled nuclei was decreased with the increase in miR-33a mimic concentrations, but increased with the increase in miR-33a inhibitor concentrations, which further supports our hypothesis that miR-33a negatively regulates myoblast proliferation (Figure 2E,F). Neither the mimic controls nor the inhibitor controls had any significant effects on the myoblast viability or proliferation.

### miR-33a targets IGF1, FST and CCND1 during myoblast proliferation

**Figure 3:**
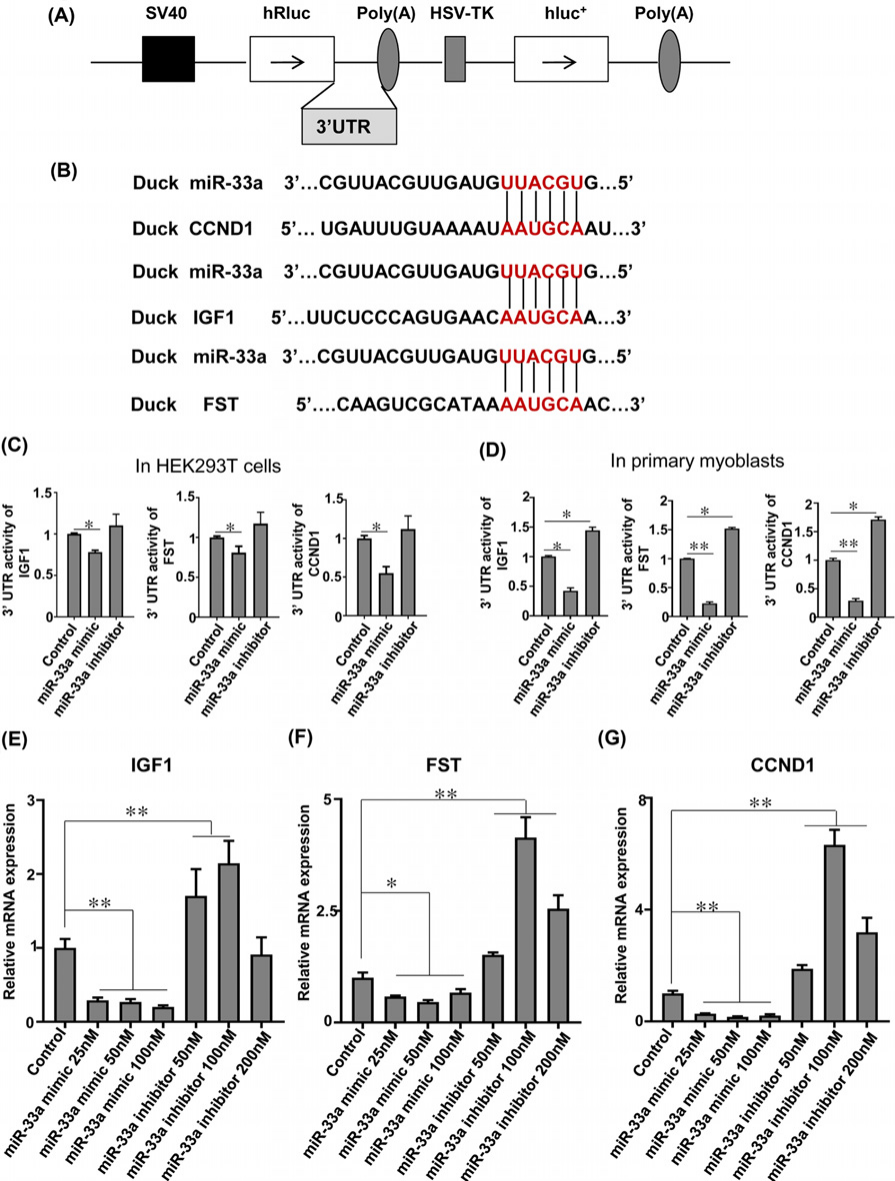
miR-33a targets IGF1, FST and CCND1 during myoblast proliferation (**A**) The schematic of the psiCHECK™-2 luciferase reporter vector. (**B**) Predicted binding sites of miR-33a in duck IGF1, FST and CCND1 3′UTR. (**C**) Overexpression of miR-33a inhibits and interference miR-33a enhances the IGF1, FST and CCND1 3′UTR reporter activity in HEK293T cell line. (**D**) Overexpression of miR-33a inhibits and interference miR-33a enhances the IGF1, FST and CCND1 3′UTR reporter activity in duck primary myoblasts; *N*=4; **P<0.01, *P<0.05. (**E–G**) Quantitative RT-PCR analysis of IGF1, FST and CCND1 expression in myoblasts after transfection miR-33a mimics and inhibitors for 24 h. Data were analyzed by the Student’s *t* test and shown as mean ± SEM; *N*=3, **P<0.01, *P<0.05.

To further investigate the mechanisms underlying the negative regulation of myoblast proliferation by miR-33a, we used TargetScan 6.2 to predicted potential miR-33a binding sites in the 3′UTR of IGF1, FST and CCND1. We generated reporter constructs expressing luciferase coding sequence fused to the 3′UTR of these genes, with an aim to assess whether miR-33a directly targets these three genes (Figure 3A,B and Supplementary Figure S2). Overexpression of miR-33a remarkably repressed the activity in 3′UTR of IGF1, FST and CCND1 both in HEK293T cells and primary myoblasts, while inhibition of miR-33a significantly enhanced the activity of 3′UTR of IGF1, FST and CCND1 in primary myoblasts (Figure 3C,D). To further determine the effects of miR-33a on the expression of IGF1, FST and CCND1, we measured the mRNA expression of these three genes (Figure 3E–G). After 24 h of transfection, overexpression of miR-33a significantly inhibited the mRNA expression of *IGF1, FST* and *CCND1*, while inhibition reduced their expression*.* A hundred nanomolar inhibitor of miR-33a significantly enhanced mRNA expression of *IGF1, FST* and *CCND1*, while 200 and 50 nanomolar inhibitor had no significant effects on *IGF1* and *CCND1* mRNA expression, respectively.

### miR-33a inhibits myoblast proliferation through the PI3K/Akt/mTOR signaling pathway

**Figure 4:**
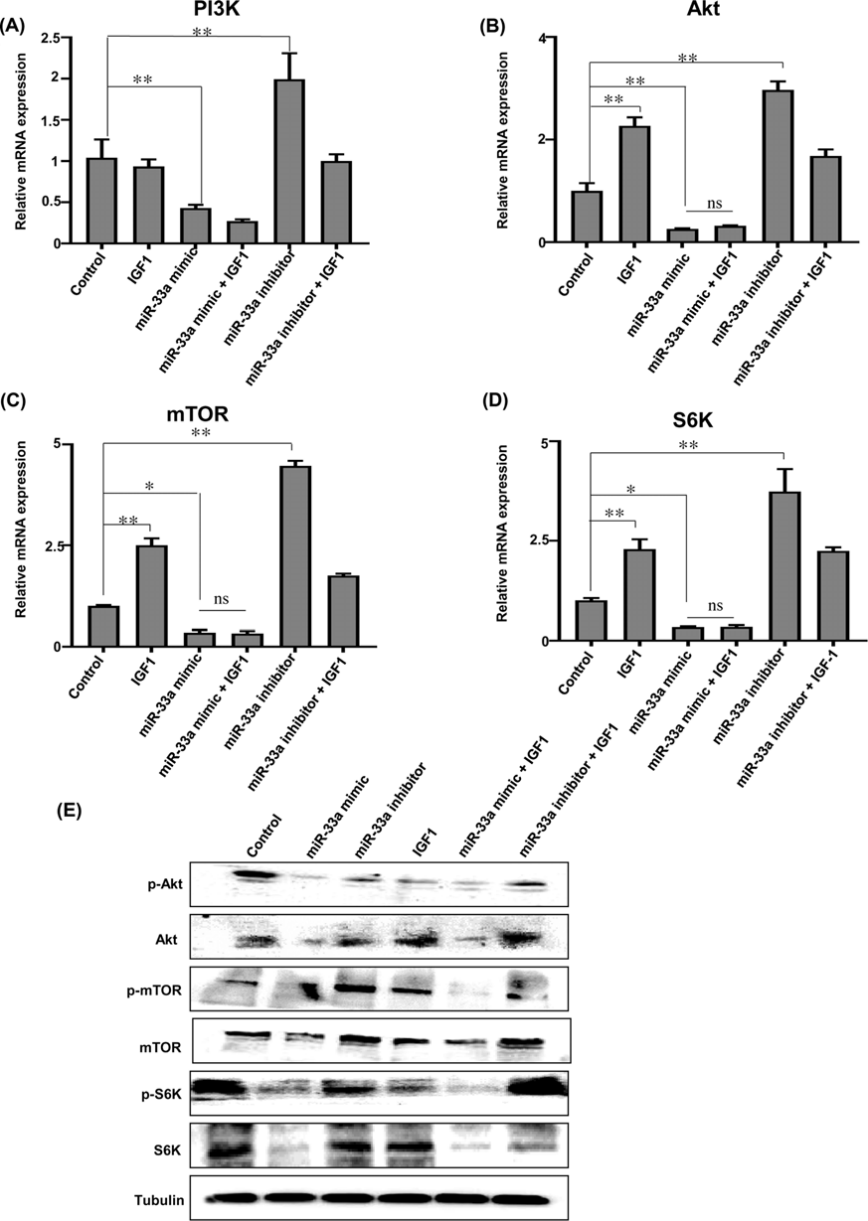
miR-33a inhibits the PI3K/Akt/mTOR signaling that located downstream of IGF1 (**A–D**) Quantitative RT-PCR analysis of *PI3K, Akt, mTOR* and *S6K* expression of myoblasts in different treatment groups. (**E**) Protein expression of Akt, phospho-Akt (Thr308) (p-Akt), mTOR, phospho-mTOR (serine 2448) (p-mTOR), S6K, and phospho-S6K (Ser417) (p-S6K) detected by Western blot after different treatments. Tubulin was used as the internal reference. Each treatment and sample was repeated three times. Data analyzed by the Student’s *t* test and shown as mean ± SEM; *N*=3; **P<0.01, *P<0.05.

IGF1 is an important growth factor in skeletal muscle development [24]. To further explore the effects of miR-33a on IGF1 function and its downstream signaling pathway, we added IGF1 to the culture medium with or without miR-33a mimic or miR-33a inhibitor. Our results showed that overexpression of IGF1 promoted the mRNA expression of *Akt, mTOR* and *S6K*, and enhanced the protein expression of Akt and p-mTOR. Overexpression of miR-33a inhibited mRNA expression of *PI3K, Akt, mTOR* and ribosomal protein S6 kinase (*S6K*), and suppressed protein expression of Akt, p-Akt, mTOR, p-mTOR, S6K and p-S6K 36 h after transfection. Interference with miR-33a increased the mRNA expression of *PI3K, Akt, mTOR, S6K* and promoted the protein expression of mTOR and p-mTOR. However, the inhibitory effects of miR-33a on the mRNA and protein expression of PI3K, Akt, mTOR and S6K were not reversed when IGF1 was added to the culture medium. When treated with miR-33a inhibitor+IGF1, the protein expression of p-S6K is increased. S6K is the downstream of mTOR, we speculate that the effect of IGF1 on S6K protein expression needs a longer time to be detectable. These results suggest that miR-33a blocking PI3K/Akt/mTOR signaling pathway through inhibiting IGF1 expression (Figure 4A–E). In our study, FST and CCND1 were also found to be the direct targets for miR-33a. Both FST and CCND1 have been reported to regulate myoblast proliferation through the PI3K/Akt/mTOR signaling pathway, but the detailed mechanisms are unclear. Activin A receptor type I (ACVR1), transforming growth factor-β2 (TGF-β2) and myostatin (MSTN) are the three competitive inhibitors of FST. Cell division protein kinase 6 (CDK6) and cell division protein kinase 8 (CDK8) are two other important genes that regulate the cell. We observed that overexpression of miR-33a increased the mRNA expression of *ACVR1* and *MSTN*, and inhibited the mRNA expression of *TGF-β2, CDK6* and *CDK8.* Interference with miR-33a increased the mRNA expression of *TGF-β2, CDK6* and *CDK8* (Supplementary Figure S3A–E).

## Discussion

miR-33a has long been known as a key negative regulator of cell proliferation. Previous studies showed that miR-33a suppressed the proliferation of many various cancer cells, such as breast cancer cells [25] and lung cancer cells [26]. However, its role in skeletal muscle development has not been investigated. In the present study, we for the first time demonstrated that miR-33a is a negative regulator of duck myoblast proliferation, and revealed the specific regulation mechanism is that miR-33a has the ability to bind to the 3′UTR specific region of IGF1, FST and CCND1, and further inhibit the PI3K/Akt/mTOR signaling pathway.

IGF1 is an important upstream regulator of PI3K/Akt/mTOR signaling pathway. It binds to insulin-like growth factor 1 receptor (IGF1R), its membrane-bound receptor, and activates the PI3K/Akt/mTOR signaling pathway, which is essential for myoblast proliferation. Several miRNAs have been implicated in the IGF1 signaling pathway. For example, miRNAs such as miR-21 and miR-432 have been demonstrated to regulate PI3K/Akt/mTOR signaling pathway during myogenesis [27,28]. miR-1 directly targets IGF1 to regulate muscle hypertrophy [29]. miR-128a, an anti-myogenic miRNA, can directly target PI3K, which is the critical gene of PI3K/Akt/mTOR signaling pathway. Akt, a member of the PI3K/Akt/mTOR signaling, was down-regulated in miR-33a-overexpressed hepatic satellite cells [13]. Our data collectively suggests that miR-33a plays key roles in regulating IGF1 and PI3K/Akt/mTOR signaling pathway. Overexpression of miR-33a inhibits not only the expression of IGF1 but also the IGF1’s downstream signaling. Specifically, overexpression of miR-33a significantly inhibit the mRNA, phosphorylation as well as the total protein expression of PI3K, Akt, mTOR and S6K. The decreased phosphorylation of Akt may be due to the decreased IGF1 expression in miR-33a overexpression treatment.

Previous studies showed that down-regulation of miR-33a promoted *CDK6* and *Proto-oncogene serine/threonine-protein kinase Pim-1* (*PIM1*) expression and induced gastric cancer cell proliferation [30], suggesting that miR-33a is a key regulator in cell proliferation. A previous investigation also showed that CCND1 could regulate the PI3K/Akt signaling pathway to affect cell growth [31]. In our study, overexpression of miR-33a significantly reduced the mRNA expression of *CCND1*. We determined that the effects of miR-33a on the inhibition of myoblast proliferation were exerted by regulation of *CDK6* and *CDK8* expression at the transcriptional level. *CDK6* and *CDK8* are specifically involved in cell proliferation [32]. Our observation that miR-33a regulates the expression of *CCND1, CDK6* and *CDK8* suggests that *CCND1* may regulate proliferation of myoblasts not only through regulating PI3K/Akt/mTOR signaling pathway but also through co-regulating CDK6 and CDK8.

Additionally, we found that overexpression of miR-33a inhibited expression of *FST mRNA*. FST is an important secretory protein and a member of the TGFβ signaling that regulates myoblast proliferation. Our previous study showed that overexpression of FST could regulate the PI3K/Akt/mTOR signaling pathway to promote the proliferation of myoblasts [20]. We can conclude that miR-33a regulates myoblast proliferation by inhibiting FST expression, which could further inhibit the PI3K/Akt/mTOR signaling pathway. We also found that overexpression of miR-33a inhibited expression of *ACVR1* and *MSTN* that are the competitive inhibitors of FST. Therefore, overexpression of miR-33a not only inhibits FST expression but also promotes expression of its competitive inhibitors to further regulate PI3K/Akt/mTOR signaling pathway and control proliferation of myoblasts.

In conclusion, our study uncovered the fundamental role of miR-33a in the inhibition of myoblast proliferation. The results suggest that miR-33a inhibits myoblast proliferation by directly targeting *IGF1, CCND1,FST* and that PI3K/Akt/mTOR signaling pathway is involved in this process (Supplementary Figure S4). Our research might provide insights into miR-33a function in overall process of skeletal muscle development and its potential therapeutic use in skeletal muscle hypertrophy and future clinical treatment for patients with skeletal muscle hypertrophy.

## Supplementary Material

Supplementary Figures S1-S4 and Table S1-S2

## Abbreviations

3′UTR: 3′untranslated region
Akt: protein kinase B
BrdU: 5-bromo-2V-deoxyuridine
CCND1: cyclin D1
CDK6: cyclin-dependent kinase 6
DMEM: Dulbecco’s modified Eagle’s medium
FBS: fetal bovine serum
FST: follistatin
IGF1: insulin-like growth factor 1
IGF1R: insulin-like growth factor 1 receptor
miRNA: microRNA
mTOR: the mammalian target of rapamycin
MyoD: myogenic differentiation antigen
PI3K: phosphatidylinositol 3-kinase
PIM1: Proto-oncogene serine/threonine-protein kinase Pim-1
PVDF: polyvinylidene difluoride
