# Supplementary material for: MicroRNA-33a negatively regulates myoblast proliferation by targeting IGF1, follistatin and cyclin D1
Source: Biosci Rep. 2020 Jun 4;40(6):BSR20191327. doi: 10.1042/BSR20191327 (PMC7273915; doi:10.1042/BSR20191327)
Supplement: Supplementary Figures S1-S4 and Table S1-S2 [file BSR-2019-1327_supp.pdf]

Supplemental Figure 1

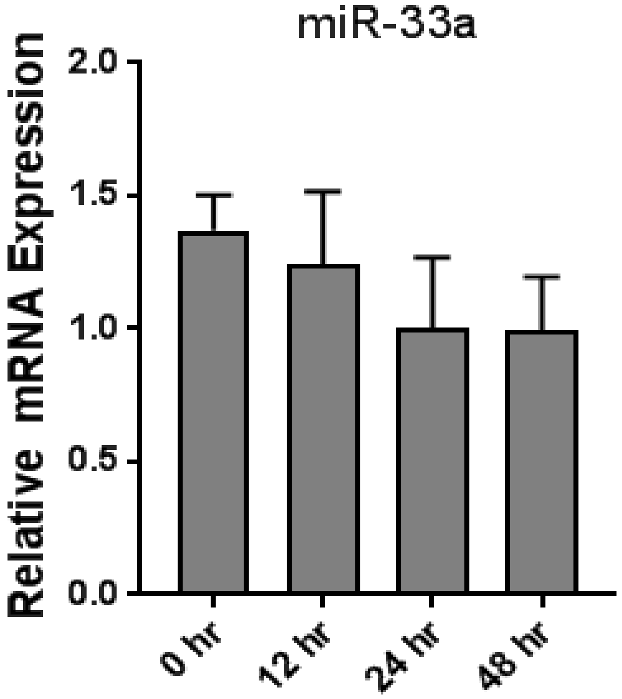

Figure S1. Quantitative RT-PCR analysis of miR-33a expression in myoblasts in 0 hr,12 hrs, 24 hrs and 48 hrs

Supplemental Figure 2

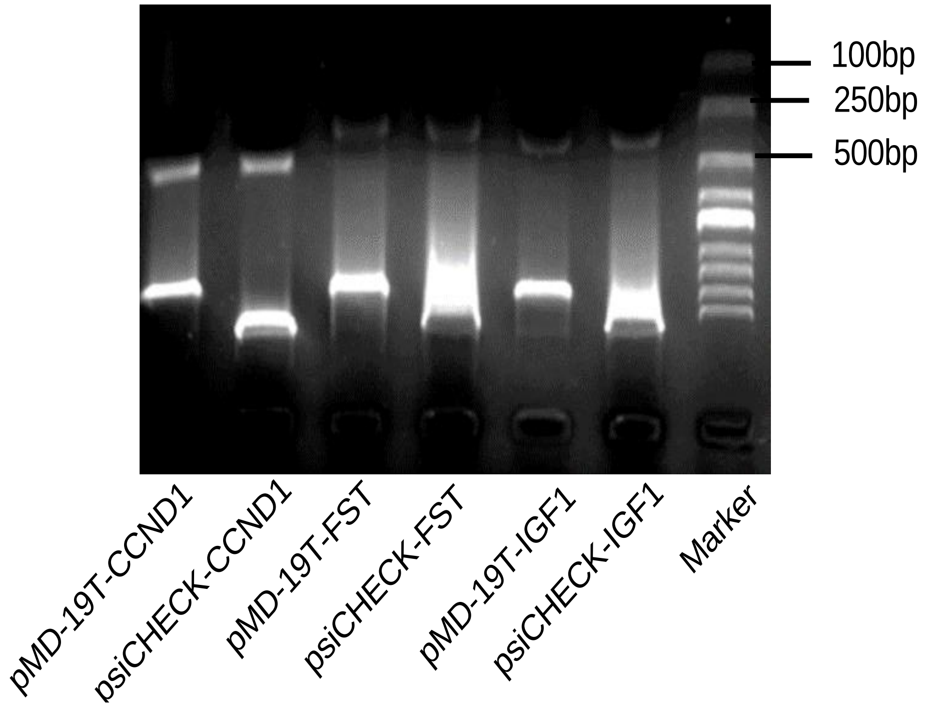

Figure S2. Identification of recombinant plasmids by double digestion

Supplemental Figure 3

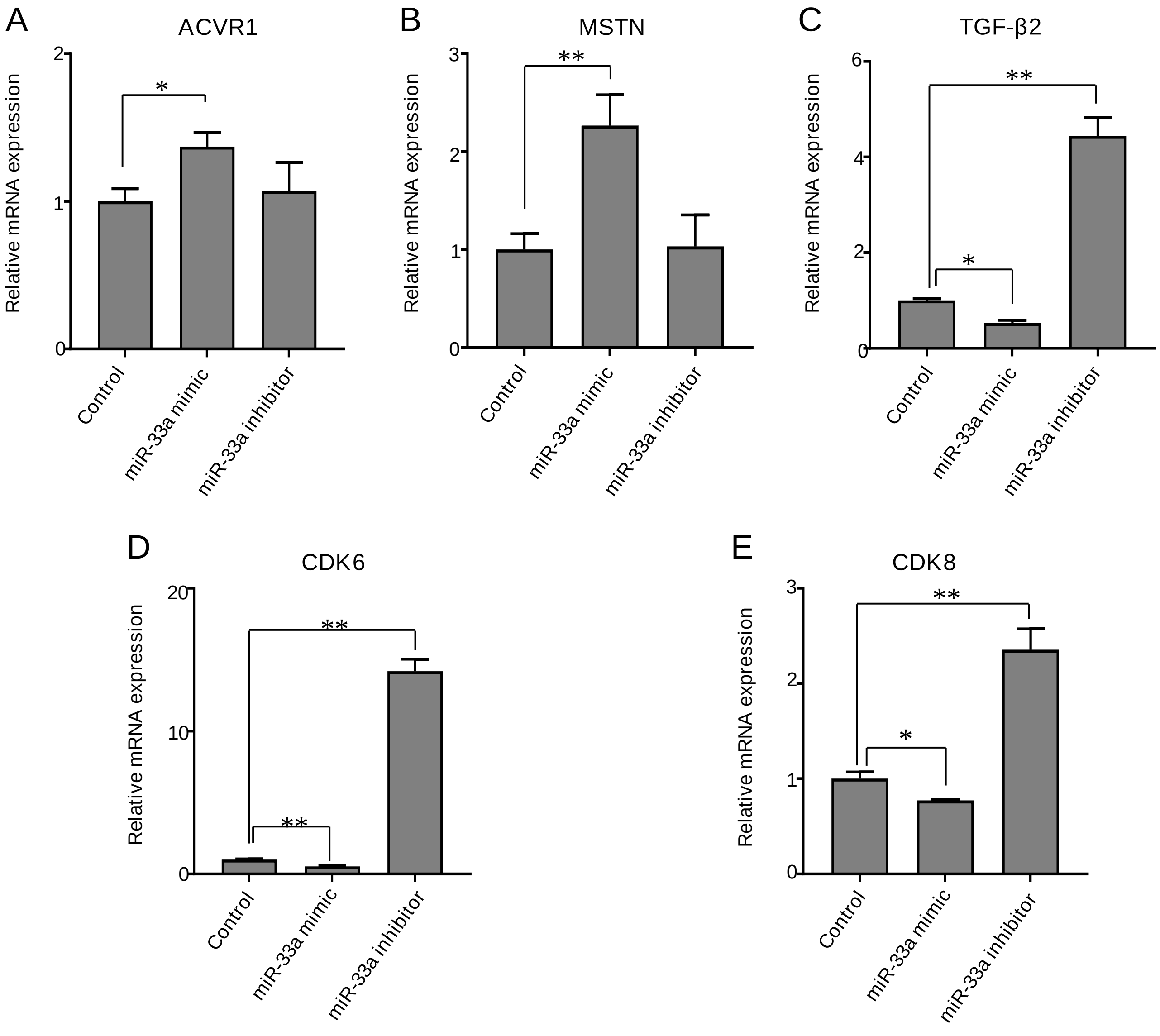

Figure S3. miR-33a inhibits the downstream signaling of FST and CCND1. (A-E). Quantitative RT-PCR analysis of *ACVR1, MSTN, TGFβ2, CDK6* and *CDK8* expression in myoblasts after transfection with miR-33a mimics and inhibitors for 24 hrs. Data were analyzed by the Student's *t* test and shown as mean ± SEM. $N = 3$;** = $P < 0.01$, * = $P < 0.05$.

Supplemental Figure 4

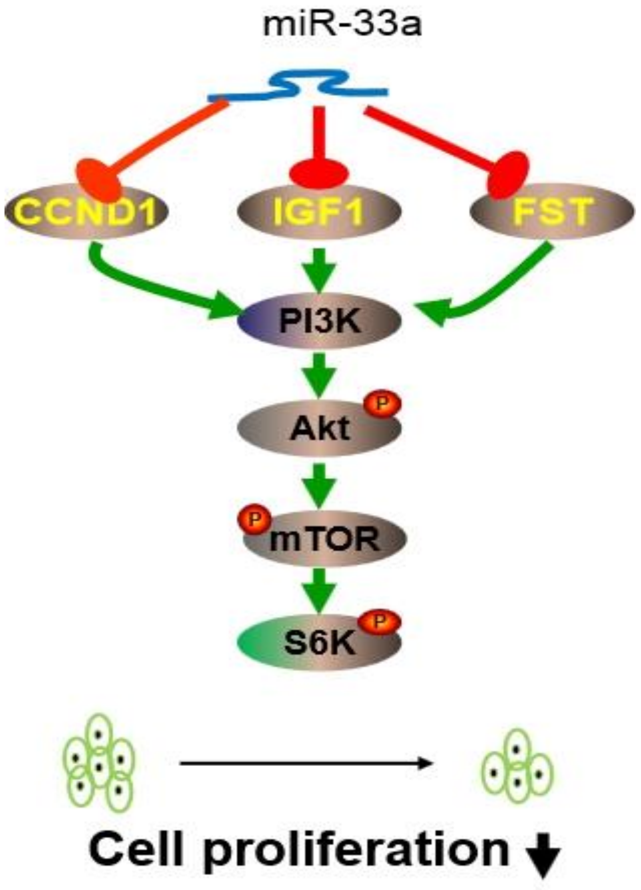

Figure S4. miR-33a regulating myoblast proliferation through PI3K/Akt/mTOR signaling pathway.

Supplemental table 1

Real-time PCR primers

Target gene	Forward Primer	Reverse Primer
FST	5'-ACAACTTACCCAAGCGAGTGTG-3'	5'-CATCTTCCTCTTCTTCCTCTGG-3'
CCND1	5'-TGGATGCCAACCTCCTCAAC-3'	5'-ACTTCTGCTCCTCGCAAACCT-3'
PI3K	5'-CTTTTACCGAGGAGGTTCTGTGG-3'	5'-CTGAAGGTTGGTCTTTGTGGAC-3'
Akt	5'-TCTTTGCTGGCATTGTTTGGC-3'	5'-GCTGTCATCTTGGTCAGGAGGAGT-3'
mTOR	5'-CTATCTGCCTCAGCTCATTCCT-3'	5'-GTCATCCAGGTTAGCTCCAAAG-3'
S6K	5'-ATAATCGTGCTGTGGACTGGTG-3'	5'-TCTGGCTTCTTGTGTGAGGTAGG-3'
MSTN	5'- GCACTGGTATTTGGCAGAGTATT-3'	5'-TCACCTGGTCCTGGGAAAGT-3'
ACVR1	5'-GGTTATGCACTCCCAAAGCAC-3'	5'-TGTAAGAGTCGAAGCAGTCAGC-3'
TGFβ-2	5'-GAAAGCCAACCACAGAGCCG-3'	5'-CCATCGCCGAGACATCAAATC-3'
CDK6	5'-TGCATCGGGACCTGAAACC-3'	5'-CAACCACTGAGGTAAGAGCCATC-3'
CDK8	5'-GCAGGGCAATAACCATACTAACG-3'	5'-TTGGAACGCTGATAGTCTGAGGT-3'
β-actin	5'-GCTATGTCGCCCTGGATTTC-3'	5'- CACAGGACTCCATACCCAAGAA-3'
GAPDH	5'-AAGGCTGAGAATGGGAAAC-3'	5'-TTCAGGGACTTGTCATACTTC-3'

Supplemental table 2

Primers for the 3'UTR amplification

Target gene	Forward Primer	Reverse Primer
FST 3'UTR	5'-ATCTGCCAATAAAACCTGAGCC-3'	5'-GAAAGCTGTAGTCCTGGTCTTCATC-3'
CCND1 3'UTR	5'-ACATATAGGACACATAGTTTAAGGATTT TC-3'	5'-ACAATCAACACCTTAGCGGCAG-3'
IGF1 3'UTR	5'-TAGAGGGAACACAGGAAACAGAAAC-3'	5'-CTCCAGCAGCCAGTGTTCATAG-3'

Supplementary Information

Supplementary Materials and Methods

Purification of RNA Using Trizol

1. Add 1 mL of Trizol per 1 × 10^6 duck myoblasts, and then dissolve the cells as quickly as possible in Trizol, solubilize the samples for 5 min at room temperature;
2. Add 0.2 mL of chloroform, mix vigorously by hand for 15 sec. Then the mixture stands for 5 min at room temperature;
3. Centrifuge at 12,000g in a centrifuge for 15 min at 4°C; transfer the upper clear phase to a fresh tube;
4. Add 0.5 mL of isopropanol of the clear phase; mix vigorously and let stand for 15 min at room temperature;
5. Centrifugation at 12,000g in a centrifuge for 10 min at 4°C to collect the precipitated RNA; then carefully remove any remaining liquid of the tube;
6. Added 1 mL 75% ethanol into the tube, then centrifugation at 8,000g in a centrifuge for 5 min at 4°C;
7. Carefully remove any remaining liquid of the tube, resuspend the RNA in RNase-free H_2O, and store at -20°C.
